# Digital Health Approaches for Improved Population Health Outcomes: Time for a Disease Vulnerability Matrix for Individuals and Communities?

**DOI:** 10.3389/fdgth.2021.720530

**Published:** 2021-09-30

**Authors:** Gaurav Laroia, Cole Zanetti, Vasant Kumar Ramaswamy, Benjamin D. Horne, David C. Klonoff, Bobby John

**Affiliations:** ^1^CareCentra, New York City, NY, United States; ^2^Ralph H. Johnson VA Medical Center, Charleston, SC, United States; ^3^Veteran Health Administration Innovation Ecosystem Director of Digital Health, Rocky Vista University College of Osteopathic Medicine, Parker, CO, United States; ^4^Cardiovascular and Genetic Epidemiology, Intermountain Medical Center Heart Institute, Salt Lake City, UT, United States; ^5^Division of Cardiovascular Medicine, Department of Medicine, Stanford University, Stanford, CA, United States; ^6^Diabetes Technology Society, Burlingame, CA, United States; ^7^Department of Medicine, University of California, San Francisco, San Francisco, CA, United States; ^8^Mills-Peninsula Medical Center, Diabetes Research Institute, Burlingame, CA, United States; ^9^Æequitas Consulting, New Delhi, India

**Keywords:** disease vulnerability, population health, precision health, health behavior, health impact bonds

## Introduction

Non-communicable diseases sit on a derangement continuum. Lifestyle behaviors and socioeconomic context in addition to the access to care, control the pace and intensity of derangement of this continuum. While pathophysiological heterogeneity has been shown to be predictive of the pace or intensity of such movement, it is opportune to propose a framework for integrated healthcare that monitors ongoing changes in vulnerability to various disease states, progression and in some cases, reversal of these states (1). A combination of data science and digital technologies can help detect the risk of derangement dynamically, while behavior science can help shape behaviors that prevent, ameliorate or reverse such risk and improve health outcomes (2). Innovative means of financing would be imperative to fund such disruptive approaches within and across economies.

### What COVID Unveiled?

The COVID pandemic has exposed what has been long suspected: Non-communicable diseases, like metabolic disorders, that present as individual organ dysfunction are vulnerability markers on a continuum of health derangement. Infections, and other stress inducers, can elicit disproportionate systemic responses when superimposed on such a derangement continuum. Current medical specialty approaches in clinical medicine often fail to see presenting symptoms and signs within this broader vulnerability context, leading to a mismatch in individual and community level health outcomes for the clinical expertise and related resources invested (3).

### The Position Within Disease Vulnerability Matrix Predicts Intervention Outcomes

To effectively manage population health, it is necessary to understand each presenting episode of a non-communicable disease as an acute-on-chronic continuum episode, to be mitigated within the context of the individual's underlying disease vulnerability.

Incorporating technology into healthcare for chronic diseases and progressive vulnerability requires an integrated approach encompassing an understanding of the disease's natural history, its markers, and its socio-economic impact when late complications can be avoided (4). This integrated approach necessitates: (1) an understanding of the natural history of chronic dysfunction, which requires treating a chronic disease before it presents as an expensive and debilitating acute disease, (2) the need for a precision medicine approach, which requires identification of multiple genetic, physiologic, environmental, socioeconomic, and behavioral markers for each patient and assignment of risks within their vulnerability matrix, and (3) the development of novel financing vehicles that will allow governments to pay for surveillance, prevention and management of chronic disease where expenses occur in the present and savings accrue in the future, and to also share in the savings with investors (5). By integrating an approach to utilizing technology that recognizes a disease's natural history, its biomarkers, and its economic impact, it will be possible to radically transform the infrastructure of healthcare delivery to more effectively manage chronic diseases, enjoy better outcomes, and save money (6).

### The Burning Platform in Managing Population Health Today

Healthcare spends by governments are not based on a zero-based budgeting exercise. The global economic slowdown as well as prioritized spending on the COVID-19 vaccine puts at risk many universal healthcare programs today. Further, the current ratio of healthcare spend is roughly 70:30 in favor of curative (i.e., spend on co-morbidities) vs. preventive spend where diseases are identified at an early stage and treated upfront. Within the curative interventions space, procedural interventions account for 80% of costs while drugs and consumables make up the rest. This necessitates the convergence of healthcare, data science, behavioral science, and technology to optimize spend and improve outcomes.

As the true scale and burden of derangements of metabolism becomes clearer within individuals, and across the population, it will become even more critical to incorporate information technology tools that are able to situate specific instances of disease in an individual within his/her vulnerability continuum when they present themselves for remedies in a healthcare setting.

### Reimagining Healthcare With Technology Solutions

Moving forward healthcare will require more precise outcome measures as governments evolve their spending toward a more balanced budget between curative and preventive care. In addition, technology and real-world evidence will be an integral part of the architecture of healthcare to ensure optimized resource utilization. These would include but not be limited to integrated solutions to improve healthcare through:

#### a. Precision Monitoring, Diagnosis, and Treatment

Risk stratification of sub-sets of population using advanced testing technologies can help predict actuarial costs for subsequent periods and triage treatment protocols based on risk levels (7). For example, AI based platforms that can leverage real world data to provide insights to physicians and health systems on the progression and acuity of co-morbidities in the case of diabetes. Identifying which diabetic patients are at a risk of dose escalation or to a basal bolus regimen can therefore be of value to health systems to manage the risk upfront to lower downstream costs.

#### b. Shaping Patient Behaviors

Research suggests that if behavior-related risk factors were eliminated, 80% of all heart disease, diabetes and stroke could be prevented, as could more than 40% of US cancer cases and deaths (8). AI/ML engines that drive a precision nudging solution to shape patient behaviors in chronic, acute, and preventative care settings can therefore significantly improve health outcomes (9). AI/ML based technologies can draw data from a variety of sources including from the participant's wearables and personal devices, surveys, and participant response patterns. These data help understand a user's motivation and ability to carry out specific health actions at any given time. Using data science and reinforcement learning techniques of AI, it is possible to design intelligent triggers or precision nudges sent via the participant's preferred channel at the right time and at the right frequency to persuade them to take tiny steps toward their health goals. Collectively, these steps help them adhere to their overall care plan including managing diet, exercise, sleep, emotional health, and prescribed care plans to achieve better health outcomes. There are very promising results from recent deployments of nudging technologies in both chronic and acute care settings within health systems in the US and Europe.

#### c. Reducing Healthcare Delivery Costs Using Combination Therapies That Include Prescription Drugs (Rx) and Digital Therapeutics

To optimally deploy digital technologies in combination with Rx therapeutics, it is important to triage diseases along a continuum- starting from intuitive approaches to medicine at one end of the spectrum to probabilistic empirical approaches, and evidence-based precision approaches at the other end. Precision care is most suited to benefit from the integration of digital technology as discrete parts of care pathways can be task shifted from high cost venues and providers of care to lower cost ones including to domiciliary settings. For example, dose titration of insulin using FDA approved dose titration apps allows glycemic control to be achieved faster with lower risk of hypoglycemia induced by patient-led modulation of insulin doses. Going forward, real-world evidence will help demonstrate ongoing value of such solutions by collecting, organizing and activating health information eventually leading to inclusion of such combination therapies in treatment guidelines (10).

### Outcomes Not Programs: Reimagining Health Financing

Financing these improvements in a redesigned healthcare delivery paradigm requires innovative approaches that incentivize health rather than treatment. In the recent past, local governments have experimented with Health Impact bonds as a way to address the cash flow challenge. These are outcome-based financing and contracting mechanism where an investor provides upfront capital to address a social problem, and then are repaid with a return by an outcome funder subject to an overall result being achieved. The attractive feature of Impact bonds is the presence of an intermediary investor who takes on the risk, makes upfront investments, and gets paid for milestones. Unlike conventional impact investments where repayment to the investor comes from program revenues, in impact bonds the repayments are made from government savings generated by an intervention. Besides the three main actors- outcome funder, investor and service provider, the impact bond involves an independent evaluator who verifies agreed upon results. A review of the medical literature on social impact bonds for non-communicable diseases was reported by Hulse et al. (5). They identified 11 Health Impact Bonds implemented in eight countries, four of which were for diabetes or diabetes related disease. They all were funded by impact investment companies providing funds to local governments as outcome payors. By linking repayment and interest to the success of the prevention program, the model was based on risk-sharing. In each case, end results of the programs have not yet been reported.

Learnings from the initial experiments with Health Impact Bonds however suggest the need for a more holistic approach and one that leverages a technology-dependent operating model for scale up. The design of the Health Impact Bond in the new context, as described above, would extensively leverage technology to converge healthcare, data science, behavioral science, and financing tools to optimize spend and improve outcomes. The deep integration of technology into the architecture of the Health Impact Bonds would also address the risk of gaming for financial incentives in SIBs due to the presence of self-reported outcomes, low rigor evaluation and low transparency. The timing for this is ripe, given the increasing emphasis of societal purpose in addition to business outcomes for companies and investors today.

## Conclusion

An integrated approach encompassing healthcare, data science, behavioral science, and technology is increasingly possible. If implemented it can address challenges to traditional healthcare that include fragmented delivery, siloed budgets, and short-term focus. The result of this historical approach to healthcare has been fragmented and reactive public services responding to crises and poor performing services going unchanged. A new lens to healthcare of each member of the population will include health programs developed with the ability to address vulnerabilities in health, such as acute flares of destabilized chronic conditions, to maintain the stability of overall population health. Empowering each individual to take charge of their health, each caregiver to focus on those in most need, and financing organizations to stand up this healthcare paradigm are implicit in the design.

## Author Contributions

All authors listed have made a substantial, direct and intellectual contribution to the work, and approved it for publication.

## Conflict of Interest

GL is a Strategic Advisor to CareCentra. VR is a co-founder of CareCentra. BJ is the founder of Æequitas Consulting. BDH is an inventor of clinical decision tools that are licensed to CareCentra and Alluceo, is the PI of grants supported by CareCentra, GlaxoSmithKline, and AstraZeneca, and is a member of the scientific advisory board of LabMe, Inc. The remaining authors declare that the research was conducted in the absence of any commercial or financial relationships that could be construed as a potential conflict of interest.

## Publisher's Note

All claims expressed in this article are solely those of the authors and do not necessarily represent those of their affiliated organizations, or those of the publisher, the editors and the reviewers. Any product that may be evaluated in this article, or claim that may be made by its manufacturer, is not guaranteed or endorsed by the publisher.
